# Porosity and distribution of water in perlite from the island of Milos, Greece

**DOI:** 10.1186/2193-1801-3-598

**Published:** 2014-10-12

**Authors:** Stephan Kaufhold, Anke Reese, Werner Schwiebacher, Reiner Dohrmann, Georg H Grathoff, Laurence N Warr, Matthias Halisch, Cornelia Müller, Ulrich Schwarz-Schampera, Kristian Ufer

**Affiliations:** Bundesanstalt für Geowissenschaften und Rohstoffe, BGR, Stilleweg 2, D-30655 Hannover, Germany; Geowissenschaften und Geographie, Georg-August-University Göttingen, Goldschmidtstr. 3, 37077 Göttingen, Germany; Knauf, Knauf Aquapanel GmbH, Kipperstraße 19, 44147 Dortmund, Germany; Energie und Geologie, LBEG, Landesamt für Bergbau, Stilleweg 2, D-30655 Hannover, Germany; Ernst-Moritz-Arndt Universität Greifswald, Institute für Geographie and Geologyie, Friedrich-Ludwig-Jahn-Str. 17a, D-17487 Greifswald, Germany; LIAG, Leibniz-Institut für Angewandte Geophysik, Stilleweg 2, D-30655 Hannover, Germany

**Keywords:** Perlite, Porosity, Water distribution, Volcanic glass, FIB, CT-analysis

## Abstract

A perlite sample representative of an operating mine in Milos was investigated with respect to the type and spatial distribution of water. A set of different methods was used which finally provided a consistent view on the water at least in this perlite. Infrared spectroscopy showed the presence of different water species (molecular water and hydroxyl groups / strongly bound water). The presence of more than 0.5 mass% smectite, however, could be excluded considering the cation exchange capacity results. The dehydration measured by thermal analysis occurred over a wide range of temperatures hence confirming the infrared spectroscopical results. Both methods point to the existence of a continuous spectrum of water binding energies. The spatial distribution of water and/or pores was investigated using different methods (CT: computer tomography, FIB: scanning electron microscopy including focused ion beam technology, IRM: infrared microscopy). Computer tomography (CT) showed large macropores (20 – 100 μm) and additionally revealed a mottled microstructure of the silicate matrix with low density areas up to a few μm in diameter. Scanning electron microscopy (FIB) confirmed the presence of μm sized pores and IRM showed the filling of these pores with water. In summary, two types of pores were found. Airfilled 20 – 100 μm pores and μm-sized pores disseminated in the glass matrix containing at least some water. Porosity measurements indicate a total porosity of 26 Vol%, 11 Vol% corresponding to the μm-sized pores. It remains unsolved wether the water in the μm-sized pores entered after or throughout perlite formation. However, the pores are sealed and no indications of cracks were found which indicated a primary source of the water, i.e. water was probably entrapped by quenching of the lava. The water in these pores may be the main reason for the thermal expandability which results in the extraordinarily porous expanded perlite building materials.

## 1 Introduction

Perlite is a hydrous volcanic material dominated by alumosilicate glass. As an industrial material, perlite is mostly used in its expanded form, i.e. after heat treatment resulting in a light-weight macroporous product. Most of the expanded perlite is used for building construction in plasters, mortars, and tiles. Minor components of perlite are phenocrysts or microlites which formed before eruption of the magma (e.g. feldspar or biotite). Technically, the term perlite is used for glassy volcanic rock which can be thermally expanded to about 20 times of its volume (Koukouzas et al. 2000). Scientifically the term perlite is used for hydrated volcanic glass. The most common volcanic glass is obsidian. The water content of typical obsidian is about 0.1 mass%. Larger water contents of obsidians mostly result from post-emplacement secondary hydration. Perlites, in contrast, contain up to 5 mass% water. They are believed to form upon hydration of volcanic glass (Ross and Smith 1955). Classical perlites (round particles with an onion like appearance) (Lorenz and Gwosdz 2000) are distinguished from banded perlites (Allen 1988). The water required for perlite formation (glass hydration) is supposed to enter through small cracks present in the volcanic glass. Diffusion of water into the glass may cause the cracks or crack formation facilitates water diffusion (Marshall 1961; Friedmann et al. 1966; Denton et al. 2009). At the wall of the cracks the glass dissolves and smectite crystallizes as alteration proceeds (Denton et al. 2009). The typical hydration shells around the primary particles are about 20 μm in size (Friedmann et al. 1966). The smectite at the walls would explain the presence of both, hydroxyls and molecular water in the perlite. Keller & Picket (1954) detected hydroxyls as well as hydrogen bound water using infrared (IR) spectroscopy. According to Friedmann et al. (1966) both primary magmatic and meteoric water causing the post-formational hydration of the glass can be found in perlites. This water, in contrast to the hydration shell, was supposed to be located within the Al-Si-framework. The difficulty of distinguishing different types of perlite water also results from the fact that the primary water of the volcanic glass varies in both amount and composition, with variable mixtures of hydroxyls and molecular water (Stolper 1982; Eckert et al. 1988; Dobson et al. 1989; Silver et al. 1990; Pandya et al. 1992).

The water in perlites can be measured by IR spectroscopy. Dobson et al. (1989) found different binding energies which could be correlated with IR stretching vibration. The amount of water is measured e.g. considering the 3550 cm^-1^ vibration (e.g. (Nichols et al. 2002)). Also NIR spectroscopy can be used (Stolper 1982). Differential thermal analysis (DTA) is commonly used for distinguishing hydroxyls and molecular water in clay minerals. However, few studies use DTA for hydrated volcanic glasses. One DTA curve was published by Tazaki et al. (1992) but the almost continuous mass decrease was difficult to interpret.

Few studies about the spatial distribution of water in perlites were published. Tazaki et al. (1992) studied freshly hydrated volcanic glasses with TEM and described spherical structures containing hydroxyls and concluded that these spherical structures could be precursors to the formation of clay minerals. Wysoczanski and Tani (2006) published IR image analysis based on the 3550 cm^-1^ vibration. They found that the glass particles all contained some water whereas low water domains corresponded to phenocrysts. However, they could not reveal the water distribution within the glass.

The present study, therefore, was conducted to improve the understanding of the spatial distribution of water in perlites. Knowledge about the spatial distribution would improve the understanding of the thermal expansion of technically produced expanded perlite.

Both, bulk methods to characterize the binding of the water (infrared spectroscopy, IR, and differential thermal analysis combined with a mass spectrometer, DTA-MS) were used along with three different 2D or 3D methods (computer tomography, CT, infrared microscopy, IRM, and focused ion beam technology in combination with a scanning electron microscope, FIB).

The perlite investigated in the present study was from the most important European perlite mines located in Milos, Greece. Most of the European perlite is derived from the Greek islands of Milos, Kimolos, and Kos (Koukouzas et al. 2000). From the middle part of the Upper Pliocene to the late Quarternary, Milos was more or less continuously affected by extensive volcanism. The perlites are supposed to result from the youngest volcanic activity in Milos which in contrast to the older ones is supposed to result from relatively shallow magma chambers (Fytikas et al. 1986). This perlite is characterized as calc-alkaline rhyolite with commonly more than 85 mass% glass and some phenocrysts. According to Koukouzas and Dunham (1994) different textural types can be distinguished: pumiceous perlite (light-weight and frothy features, only present in mines), hard perlite (dense and reddish and only appearing in the Kerdari Cap), and classical perlite (dense and mostly in contact with the hard and the pumiceous perlite in the mines). Much is known about the geological history of Milos and some information about compositional variation of Milos perlites and their applicability is available (Koukouzas et al. 2000) but none about the spatial distribution of the water within the perlite.

## 2 Materials and methods

The perlite investigated in the present study was collected in a running perlite mine in Milos (Greece). Three different specimens representing three different regions in the outcrop were collected. They differed with respect to their color (reddish, cream, white). However, no difference in water content or type of water (measured with DTA-MS and IR) was detected. Therefore, each of them would have been suitable for studying the spatial distribution of water of perlite of this deposit. One of the cream white particles (about 3 cm^3^) was selected for producing a polished thin section. This particle (the part not used for the production of the thin section) was further used for the investigation of the spatial distribution of water. For the computer tomography (CT) investigation a small piece (ca. 1 mm^3^) was cut off. In addition an expanded 1 mm^3^ sized perlite specimen (again about 1 × 1 × 1 mm = 1 mm^3^) that was taken from an industrially produced product (thermally expanded perlite) was investigated with CT.

For the characterization of the bulk material, three particles with the different colors were ground together to represent the entire outcrop.

The methods used were selected to i) characterize the bulk material and ii) to gather information about the spatial distribution of pores and or water. The latter was thought to be difficult because of expectedly small pores and/or water domains. Therefore, apart from light microscopy, methods with higher resolution and different strengths were used.

### Bulk material characterisation

X-ray diffraction (XRD) patterns were recorded using a PANalytical X’Pert PRO MPD Θ-Θ diffractometer (Cu-Kα radiation generated at 40 kV and 30 mA), equipped with a variable divergence slit (20 mm irradiated length), primary and secondary soller slits, a Scientific X’Celerator detector (active length 0.59°) and a sample changer (sample diameter 28 mm). The samples were investigated from 2° to 85° 2Θ with a step size of 0.0167° 2Θ and a measuring time of 10 sec per step. For specimen preparation, the top loading technique was used and quantification performed based on Kaufhold et al. (2010).

The chemical composition of powdered samples was determined using a PANalytical Axios. Samples were prepared by mixing with a flux material (Lithiummetaborate Spectroflux, Flux No. 100A, Alfa Aesar) and melting into glass beads. The beads were analyzed by wavelength dispersive X-ray fluorescence spectrometry (WD-XRF). To determine loss on ignition (LOI) 1000 mg of sample material was heated to 1030°C for 10 min.

For measuring mid (MIR) infrared spectra the KBr pellet technique (1 mg sample/200 mg KBr) was applied. Spectra were collected on a Thermo Nicolet Nexus FTIR spectrometer (MIR beam splitter: KBr, detector DTGS TEC). The resolution was adjusted to 2 cm^-1^.

Thermoanalytical investigations were performed using a Netzsch 449 F3 Jupiter thermobalance equipped with a DSC/TG sample holder linked to a Netzsch QMS 403 C Aeolus mass spectrometer (MS). 100 mg of powdered material previously equilibrated at 53% relative humidity (RH) was heated from 25–1100°C with a heating rate of 10 K/min.

The cation exchange capacity (CEC) was measured using the Cu-Triethylenetetramine method (Meier and Kahr 1999; Kaufhold and Dohrmann 2003). A sample mass of 0.3 and 0.4 g was used to increase the smectite detection limit up to about 0.5 mass%.

The porosity was determined based on measuring the particle or specific density (AccuPyc 1330 of micromeritics using He) and bulk or envelope density (micromeritics GeoPyc 1360 using a free-flowing, finely divided, dry powder (DryFlow®) as the fluid medium instead of a liquid with a lower diameter limit of 50 μm). Different types of samples were investigated: powder, 1–2 mm particles, >2 mm, and after melting the sample (1150°C for 6 hours).

### Methods to gather spatial information

Water can be detected by its characteristic infrared vibrations and the spatial distribution can be probed by IR-microscopy. In the present study a Thermo Nicolet Continuum FT-IR microscope was used. A freshly broken even surface of the perlite was fixed on the x-y stage. The following experimental conditions were selected: beam splitter CaF_2_, detector MCT/A, aperture 5, spectroscopic range of each spectrum 1000 – 4000 cm^-1^ with a resolution of 4 cm^-1^ and 16 scans each, and the step width was 3 μm. Because of the typically low intensity at large wave length the water deformation band at about 1635 cm^-1^ was selected to produce a 2-D plot.

The micro-computed tomography (μ-CT) imaging was performed with an “nanotom s 180” device, developed by GE Sensing & Inspection Technologies and using the product line of phoenix x-ray. This CT has a special high power nanofocus tube (180 kV/15 W) with an adjustable focal spot size down to 1 μm in diameter, which enables very sharp imaging data sets. After the scanning process, the 3D data sets have been evaluated with VG Studio Max 2.0. Phase segmentation has been performed by using quantification tools, such as edge detection and phase contrast filter operations, to ensure high accuracy phase thresholding and volume determination. Afterwards, 2D as well as 3D visualization of regions of interest within the samples took place. The scanning parameters for the investigated samples were voltage 55 kV, current 80 μA, projections 1500, average 7, skip 3, timing 1000 ms and voxel size 1.06 μm.

For the high-resolution SEM focused ion beam (FIB-SEM) investigation a Zeiss Auriga field equipped with a field emission cathode and extra-large charge coupled device (CCD) 80 mm^2^ CCD detectors for energy dispersive x-ray (EDX) analyses was used. Before starting the milling process the sample was sputtered with Pd. A “slice-and –view” procedure was run by milling 25 nm thick cuts in the form of a cross-section 20 × 20 μm in size. A full description of the methodology is described in Warr and Grathoff (2011).

## 3 Results

### 3.1 Basic characterization of the perlite

**Figure 1:**
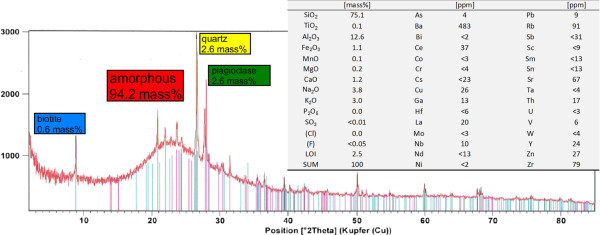
**Mineralogical and chemical composition of the perlite sample.**

The representative perlite sample consisted mainly of silicate glass (“amorphous” in Figure 1). Minor components were biotite, quartz, and feldspar. The material can be characterized as peraluminous rhyolite glass with biotite, quartz, and feldspar phenocrysts. The most abundant element was Si (75 mass% SiO_2_), followed by Al_2_O_3_ and minor amounts of the common earth and alkaline metal oxides. The chemical composition along with the LOI were determined after drying of the sample at 105°C. Hence, externally bound water is not included in the LOI value given in Figure 1. The mineralogical composition was quantified using the XRD Rietveld technique, which confirmed the presence of more than 90 mass% of an amorphous phase and about 5 mass% minor crystalline components (2.6 mass% quartz, 2.6 mass% feldspar, 0.6 mass% biotite). SEM-EDX investigations and light microscopy of the polished section revealed some additional minor components, namely apatite, zircon, hornblende, and ilmenite, which were below the XRD detection limit.

The cation exchange capacity was practically 0 meq/100 g. In none of the 6 separate measurements any systematic reduction of the extinction at 578 nm was detected. The smectite content, therefore, is less than 0.5 mass%.

**Figure 2:**
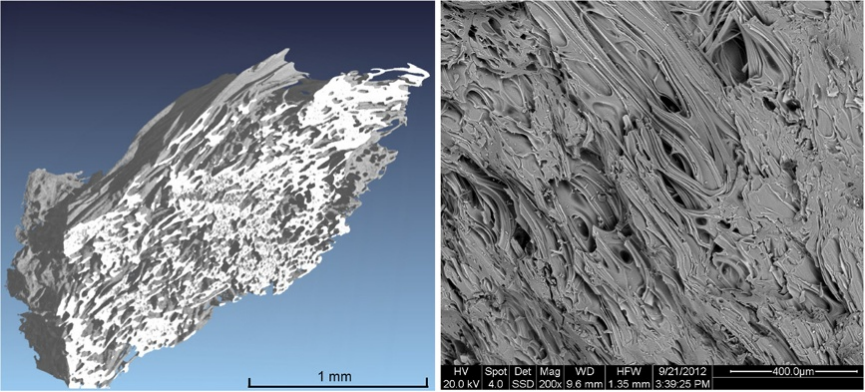
**Microstructure of raw perlite visualized by CT (left) and SEM (right).**

**Figure 3:**
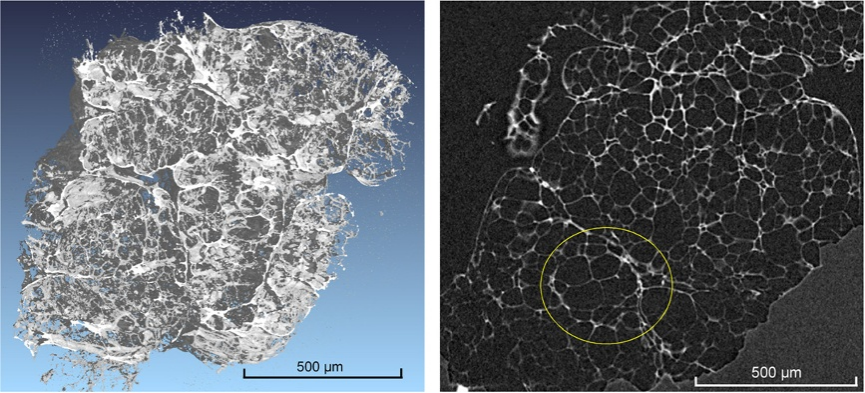
**Microstructural investigation of expanded perlite by CT (left pseudo 3D image, right 2D cross-section).** The circle shows that small pores with thinner walls are included in spherical structures with somewhat larger walls.

The microstructure of the perlite was investigated by SEM and CT (Figure 2). The low density of the perlite results from the extensive network of pores between 20 and 100 μm. The significantly higher porosity of the industrially expanded perlite was imaged. The pore walls of this material are comparably thin. The section through the expanded perlite shows a hierarchy of different silicate skeletons (Figure 3, right). Mostly clusters of smaller pores are arranged in a silicate skeleton with slightly thicker walls (circle in Figure 3).

### 3.2 Characterization of the water binding

**Figure 4:**
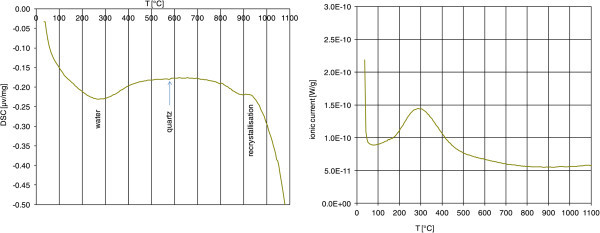
**DSC curve (left) and MS-H**_**2**_ **O curve (right) of the perlite sample.**

To investigate the binding energies of the water, thermal analysis and IR measurements were conducted. Thermal analysis showed a dehydration peak ranging from approximately 100°C up to 400°C (Figure 4) with a peak slightly below 300°C. This peak was observed in the DSC curve - with increasing temperature with the negative values reflecting the endothermal dehydration reaction and the upward peak indicating an exothermal reaction such as recrystallization occurring at ca. 930°C - as well as in the water mass spectrometer (MS) curve (mass 18). Other natural water containing materials such as swelling clays or even zeolites typically show much sharper dehydration peaks i.e. a water desorption within a smaller temperature range. The small DSC-peak at 573°C resulted from the quartz inversion. The amount of water lost from 100 to about 600°C was 2.7 mass% (read from the TG curves, not shown here) which is in reasonable agreement with the LOI data shown before.

**Figure 5:**
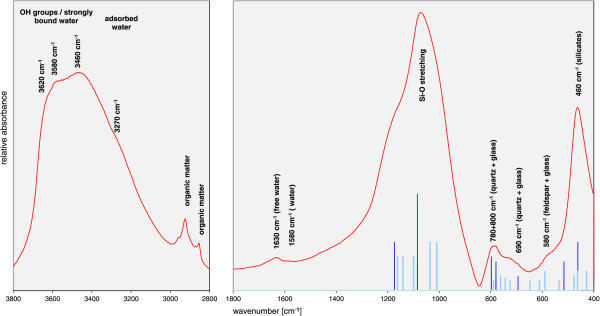
**IR spectrum of the perlite sample (pellet dried for 24 h at 150°C in a vacuum oven).**

The MIR spectrum after drying for 24 h at 150°C in vacuum still showed the presence of molecular water (1630 cm^-1^ + intensity around 3400 cm^-1^) along with the typical Si-O and Al-O-Si vibrations resulting from the crystalline and the amorphous silicates (Figure 5). In the OH stretching region no distinct band could be found. Instead a broad band ranging from the typical OH stretching region of Al-rich clay minerals (±3630 cm^-1^) to the region being typical of water adsorbed in micropores (e.g. 3410 cm^-1^ in the case of ferrihydrite) was observed. The DTA curve could be interpreted in a way that the water inside the silicate glass could not leave at lower temperatures, which would shift the dehydration towards larger temperatures. However, such kinetic effects do not affect the IR spectrum. Therefore, both, the IR and DSC results, indicate the presence of a continuous range of differently bound water molecules ranging from structural OH groups similar to those present in clay mineral structures to free molecular water. The bands at larger wavenumbers (e.g. 3620 cm^-1^) can also result from water bound directly to cations (strongly bound to a surface as inner sphere complex (Bishop et al. 1994)).

### 3.3 Spatial information

#### Light microscopy

**Figure 6:**
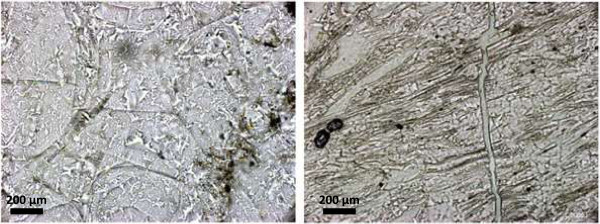
**Light microscopical analysis of the thin section of the perlite sample.**

Light microscopy was performed to find fluid inclusions which cannot be detected by SEM or CT methods. Both types of perlite texture, the classical texture (Figure 6, left) and the banded type of perlite (Figure 6, right), were observed. Light microscopy also revealed an extensive network of cracks which could have been the pathway for water which hydrated the volcanic glass.

#### Computer tomography (CT)

**Figure 7:**
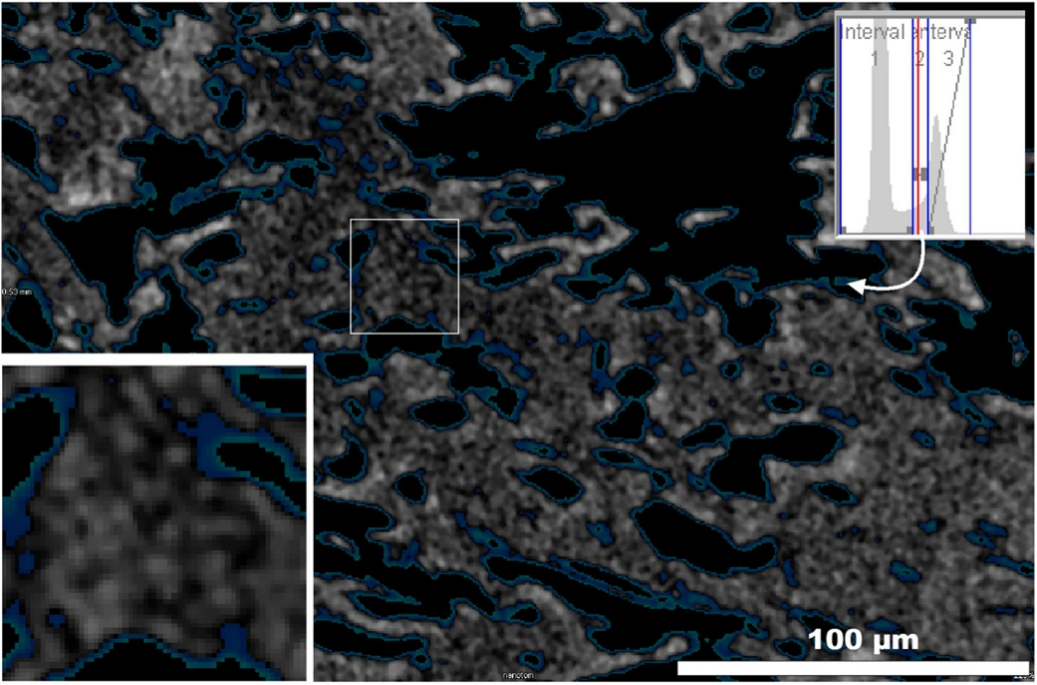
**Grey value analysis of a CT section and magnified detail of the image.** The top right insert shows the grey value distribution over the entire sample.

CT allows investigation of the 3D structure of a sample and provides 2D sections. The grey values of the image shown in Figure 7 (1 μm thick 2D section) correspond to the density of the investigated particles. A mottled structure was observed within the glass particles. Hence even the interior of the volcanic glass is not as homogeneous as expected. Disseminated dark spots were observed which indicated a possibly existing porosity. The diameter of these spots was 1–4 μm. The spots were dark because they were transparent in the 1 μm section due to water or gas filling.

In addition an automatic grey scale analysis was performed. Most of the detected grey values were black (corresponding to interval 1 in the top right insert in Figure 7). The second most grey value was attributed to the volcanic glass (interval 3). However, a closer look at the interval 3 peak reveals a shoulder towards slightly lower density grey values. These could be the hydrated walls of the glass surface or otherwise represent an artifact. This grey value interval 2 was selected and the corresponding areas were marked in blue (Figure 7). These particles or areas are almost entirely located at the particle edges indicating the beginning of hydration of the outer surfaces of the glass particles.

#### FT-IR-microscopy

**Figure 8:**
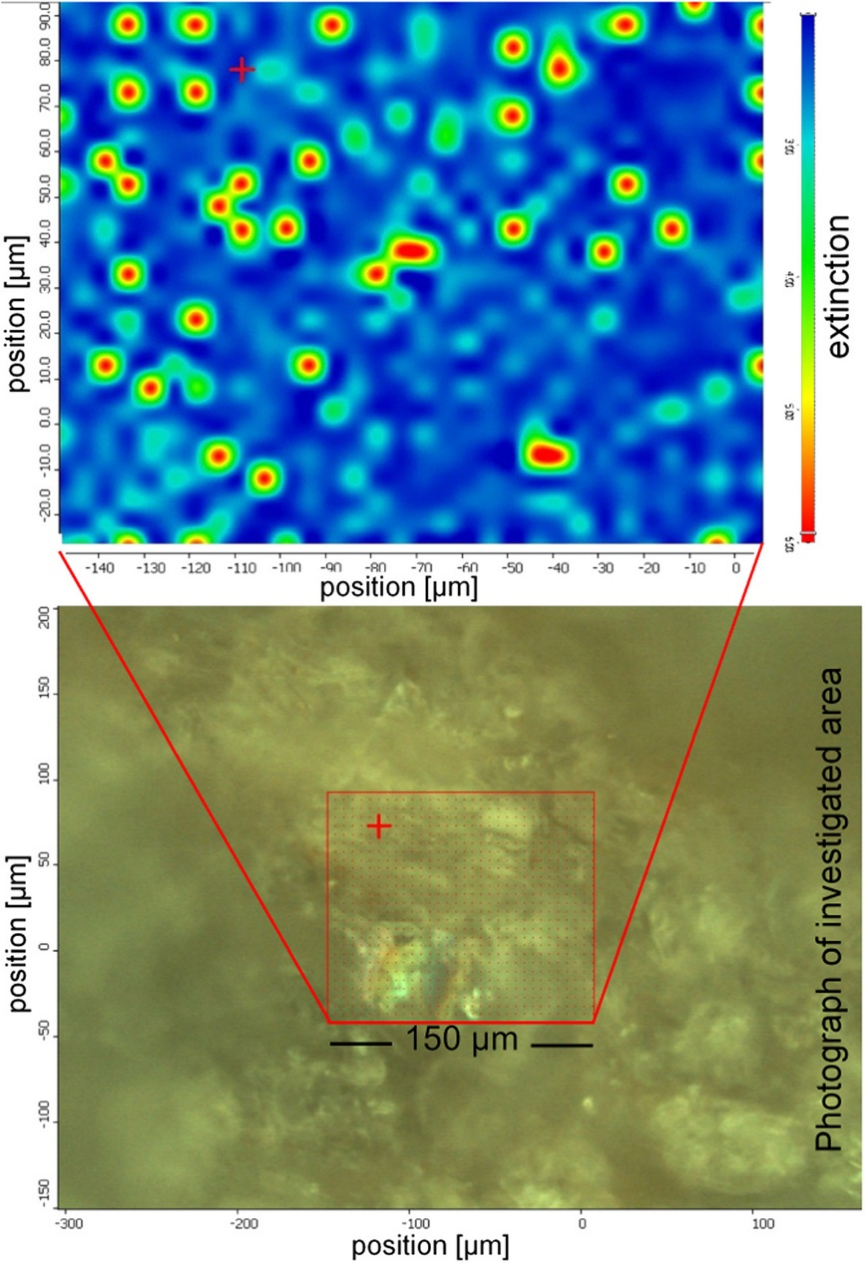
**Infrared microscopy of the perlite sample.** Top: 2-D plot of the extinction of the 1635 cm^-1^ vibration of water, bottom: photograph of the perlite surface and investigated area.

The investigated area of the even surface (unpolished) was approximately 150 μm × 100 μm (Figure 8). Altogether more than 2000 spectra were collected across this area. The individual spectra were dominated by the Si-O-stretching bands. Because of the reflectance mode the quality of the spectra was poor but differences in the water deformation region around 1635 cm^-1^ were observed. After normalization of all spectra the extinction of this band was plotted as 2-D image (Figure 8). This image proved the presence of water in several small areas with a diameter of about 5 μm. These domains appear circular, which is an artifact caused by the resolution of the microscope which is not sufficient to detect the shape of the water domains. Nevertheless, the IR-microscopy showed the presence of water in these small domains.

#### FIB-SEM

**Figure 9:**
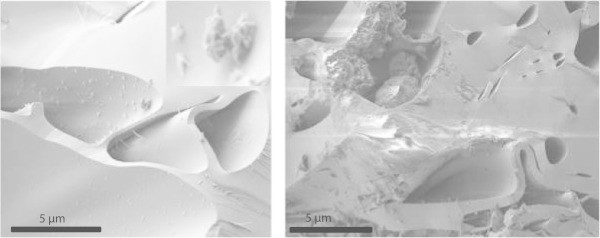
**SEM (high-vacuum) image of some pores of the perlite showing indications for hydration products.**

Crossbeam FIB-SEM investigations carried out in the high-vacuum mode provided much clearer SEM images of the glass and of possible hydration products. In the left image of Figure 9 small sub-micron sized flakes were observed. These could be dust but they were evenly distributed over the surface and showed a smectite like morphology (the typical rose like aggregates). In the right image of Figure 9 a coating of some parts of the surface was observed which again considering the morphology could be clay minerals. These phases could be smectites or other hydration products of the volcanic glass. However, the content of these clay minerals is much below the detection limit of the XRD and CEC method as discussed before. They can only be found on a few surfaces and hence they are not important from a statistical point of view.

**Figure 10:**
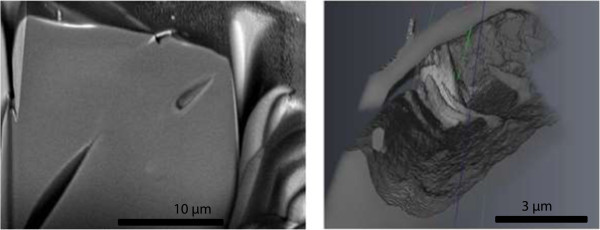
**SEM (high vacuum) image of FIB slice 107 (left) and AVIZO Fire-visualization (3D slice and view reconstruction) of one of the pores based on the FIB measurements (right, image width 10 μm).**

The 2D polished slices and “slice-and-view reconstructions” (Figure 10) show the size and shape of two of the pores and potential alteration rims around the pore as well as minor heterogeneities in the glass (Figure 10). The back scatter electron (BSE) images revealed pores coated by higher density glass, which may be partly an artifact, based on that some of the rims contain Gallium derived from the milling processes. Using the 3D-visualisation software AVIZO Fire (http://www.vsg3D.com) one of the pores was investigated in more detail (Figure 10, right). Some columnar structures were observed in the pore.

#### Porosimetry

The porosity was determined by He- and Dryflow®-pycnometry. The density of the glass without any pores (after melting) was about 2.6 g/cm^3^ a typical value of silicate glasses. Without extensive heat treatment and grinding the μm-sized matrix porosity was thought to be intact but He could of course - only - enter the larger macropores. The specific density of this material was about 2.3 g/cm^3^. This difference, therefore, can be explained by the existence of the μm-sized matrix-porosity accounting for 11 Vol%. The Dryflow®-density of the larger grain fraction was 1.8 g/cm^3^ which results in a total porosity of about 26 Vol%. This value is supposed to include the macropores. The porosity of the silicate matrix is about 10%.

## Discussion

Infrared spectroscopy showed extinction over a wide range of wavenumbers covering both the OH-stretching region of clay minerals as well as the spectral region typical of free water. This indicated the presence of a range of water binding forms which is in accordance with Dobson et al. (1989). As a further tool to study water binding, differential thermal analysis was applied which confirmed the IR results. The MS-H_2_O curve (evolved gas analysis measured by mass spectrometry) showed dehydration over an unusually broad temperature range (from 100 to 400°C) which was already observed by Tazaki et al. (1992). Desorption of free water is commonly observed between 100 and 200°C well separated from dehydroxylation (500–750°C). However, no separate second peak which could be assigned to dehydroxylation was observed. Instead one broad peak was found which may correspond to both dehydration and dehydroxylation. This conclusion is supported by the IR results which also indicated the presence of a range of different water bindings, ranging from the typical hydroxyl stretching vibrations to adsorbed water. The hydroxyls do not result from smectite because the smectite content as determined by the cation exchange capacity (CEC) method was ≤0.5 mass%. This content was too low to conclude that the hydroxyls were associated to smectite. One explanation would be that the walls of an extensive pore system are covered with hydroxyls or at least strongly bound water which in terms of binding energy are similar to clay mineral hydroxlys but in this case only cover the very surface.

Using the thermal gravimetry curve the water liberated from the perlite could be quantified (about 2.7 mass%). This value was slightly larger than the loss on ignition (LOI = 2.5 mass%) which can be explained by the fact that the sample was stored at 53% r.H. prior to thermal analysis but dried at 60°C prior to LOI determination. Both values, however, are in good agreement.

**Figure 11:**
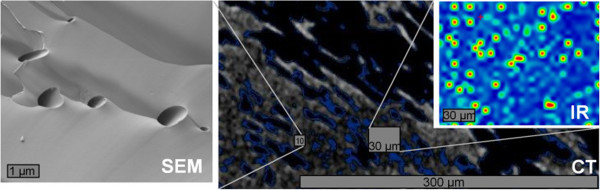
**Concluding figure showing the mottled like microstructure revealed by CT, the filling of pores with water (IR), and the presence of small pores detected by the SEM (FIB).**

The aim of the present study was to investigate the spatial distribution of the water. Different methods were used, each with specific strengths and weaknesses. Computer tomography provided a 3D image of density distributions. Using image analysis a grey value distribution was calculated which showed slightly darker surfaces of the particles which indicated areas of lower density, probably a beginning hydration. As explained above, the smectite content was too low to explain this observation. It could be explained by a small hydration layer but further interpretations would be speculative. The most interesting CT-results were found by considering 1 μm slices (2D assessment of the 3D data set). Of course the large pores which were observed with light microscopy were well resolved. In addition, however, the massive glass particles appeared uneven. Dark spots close to the resolution of the method were observed all over the glass particles. Analytical artifacts were excluded and the hypothesis was that these areas could be pores. To further investigate this assumption high resolution scanning electron microscopy in combination with the focused ion beam technology allowing 3D analysis was used. This method confirmed the presence of pores in the μm range and hence supported the view that the density heterogeneity measured by CT corresponded to a system of pores. FIB-SEM proved that the pores are closed and isolated. Neither CT nor FIB were suitable to determine eventual pore filling. Therefore, IR microscopy was used. The extinction at 1635 cm^-1^ over a 150 × 110 μm area showed spots indicating water. The shape of these spots was an artifact because of the maximum resolution of method. However, IR microscopy proved the presence of water in small domains. At first sight these domains were considered to be analytical artifacts. However, the size and distribution of these domains fits well with the pores found by CT and FIB. Therefore, these domains are supposed to correspond to the pores observed by CT and FIB and, moreover, identified at least a part of the filling of the pores. To show that the three methods probably identified the same pores all results were plotted in one figure (to be able to compare the scale; Figure 11).

The total porosity was 26 Vol% and the μm-sized matrix-porosity accounted for 11 Vol%. IR microscopy showed that the pores are at least partially filled with water. A 3D-FIB visualization of one pore showed columnar structures in the pore. These could be precipitations or newly formed phases. Further discussion about them would be highly speculative.

Most of the perlites are supposed to have formed upon post-emplacement hydration. The μm-sized finely distributed pores identified in the present study, however, are ubipresent in the volcanic glass and the pores are not supposed to be connected. The pores observed are all closed. One option to explain the abundance of this water is that it was trapped when the magma was quickly cooled. This had to be investigated further, e.g. using isotope methods.

## Summary and conclusions

Perlites are believed to form upon post-formational hydration often leading to smectite formation. The presence of smectite in the investigated sample, however, can be excluded. CEC measurements with high sample masses are accurate enough to prove that less than 0.5 mass% smectite was present. Infrared spectroscopy and thermal analysis showed the presence of a continuous range of water binding, ranging from hydroxyls/strongly bound water to molecular water. Using CT and FIB an extensive pore system of closed pores was found with pore diameters in the range of 1–5 μm. In the same range, infrared microscopy revealed domains with significant extinction in the water deformation region. Because of similar size and distribution these signals were believed to represent the filling of the pores. According to the consistent picture gained from applying a set of different methods, the glass particles of at least the investigated material contain appreciable small water filled pores. It remains unsolved wether the water in these pores entered after or throughout the emplacement. However, the pores are sealed and no indications of cracks were found which indicates a primary source of the water, i.e. water was probably entrapped by quenching of the lava. The water in these pores may be important for the possible formation of clay minerals out of perlites and may have implications for the formation of bentonites in Milos. The water in the μm-sized pores may be the main reason for the thermal expandability of this perlite.
